# CD8^+^ T cells predicted the conversion of common covid-19 to severe

**DOI:** 10.1038/s41598-021-81732-4

**Published:** 2021-01-26

**Authors:** Li Liu, Zhiyong Chen, Yingrong Du, Jianpeng Gao, Junyi Li, Tiqin Deng, Chen Chen, Lin Wang, Yongrui Yang, Chunyun Liu

**Affiliations:** 1 Department of Liver Disease, The Third People’s Hospital of Kunming City, Yunnan, China; 2 Department of Infectious Diseases, The First People’s Hospital of Zaoyang City, Hubei, China

**Keywords:** SARS-CoV-2, Viral infection

## Abstract

To evaluate the predictive effect of T-lymphoid subsets on the conversion of common covid-19 to severe. The laboratory data were collected retrospectively from common covid-19 patients in the First People's Hospital of Zaoyang, Hubei Province, China and the Third People's Hospital of Kunming, Yunnan Province, China, between January 20, 2020 and March 15, 2020 and divided into training set and validation set. Univariate and multivariate logistic regression was performed to investigate the risk factors for the conversion of common covid-19 to severe in the training set, the prediction model was established and verified externally in the validation set. 60 (14.71%) of 408 patients with common covid-19 became severe in 6–10 days after diagnosis. Univariate and multiple logistic regression analysis revealed that lactate (*P* = 0.042, OR = 1097.983, 95% CI 1.303, 924,798.262) and CD8^+^ T cells (*P* = 0.010, OR = 0.903, 95% CI 0.835, 0.975) were independent risk factors for general type patients to turn to severe type. The area under ROC curve of lactate and CD8^+^ T cells was 0.754 (0.581, 0.928) and 0.842 (0.713, 0.970), respectively. The actual observation value was highly consistent with the prediction model value in curve fitting. The established prediction model was verified in 78 COVID-19 patients in the verification set, the area under the ROC curve was 0.906 (0.861, 0.981), and the calibration curve was consistent. CD8^+^ T cells, as an independent risk factor, could predict the transition from common covid-19 to severe.

## Introduction

Since December 2019, some cases of unexplained pneumonia had been reported in Wuhan City, Hubei Province. Finally, the pathogen that caused the outbreak was initially identified as a novel coronavirus. On January 12, 2020, the virus was named as 2019 novel coronavirus (2019-nCoV) by the WHO^1^. The symptoms caused by the new coronavirus disease (COVID-19) could be mild or severe, and even some patients without any symptoms. Early recognition is extremely important for controlling the prevalence and spread of the disease^2,3^.

The incubation period after 2019-nCoV infection was 2–14 days, mostly 3–7 days. Most patients with COVID-19 showed fever with or without respiratory symptoms in the early stage of the onset. Respiratory symptoms were mainly dry cough; a small number of patients had fever or not obvious at the onset of illness, or even without fever, only dry cough. Severe patients gradually developed respiratory failure, and even acute respiratory distress syndrome and (or) shock^4^. In clinical work, it had been observed that the symptoms and signs of most light and ordinary patients had been very stable, and some patients suddenly became severe or critical in the course of 7 to 10 days and had to be transferred to ICU for treatment.

The prediction of prognosis and risk factors of severe and critically patients had been seen in relevant studies^5–7^. However, the risk factors and predictions of the transition from light or ordinary patients to heavy or critical have not been reported. We had collected clinical data from these patients in order to analyze the clinical symptoms, laboratory and imaging characteristics of these 2019-nCoV infected patients, especially the characteristics before the transition from light and normal to heavy and critical, and established a predictive model to provide a evidence for predicting changes of the disease.

## Materials and methods

The study had been approved by the medical ethics committee of the Third People’s Hospital of Kunming city, Yunnan and The First People’s Hospital of Zaoyang city, Hubei, which conformed to the principles of Helsinki declaration. From January 20, 2020 to March 15, 2020, patients with covid-19 diagnosed by The third people’s hospital of Kunming city, Yunnan and The first people’s hospital of Zhaoyang city, Hubei were collected. The specific data were collected including demographic characteristics, basic diseases, clinical symptoms and signs, clinical data, laboratory test data, chest CT and clinical outcome and so on. 408 patients with covid-19 from the first people's Hospital of Zaoyang City in Hubei Province were taken as the training set, 408 patients were all mild or normal at the time of diagnosis, and 60 patients changed to severe or critical during the treatment process, which was used to construct a predictive model; 78 patients with covid-19 from the third people's Hospital of Kunming City in Yunnan Province as the verification set, 78 patients were mild or normal at the time of diagnosis, and 14 cases were converted to severe or critical during the treatment process to verify the effectiveness of the model. Those who were severe or critical at the time of diagnosis were excluded. The endpoint variable evaluated was the transition from mild or common patients to severe or critical. The informed consent was obtained from all the participants or, if subjects were under 18, from a parent and/or legal guardian, died patients from legal guardian.

According to novel coronavirus pneumonia diagnostic criteria^8^: (1) epidemiological history: 14 days before onset, there were travel history or residential history in Wuhan and other surrounding areas or other reported cases; There was a history of contact with new coronavirus infected persons (positive for nucleic acid detection) within 14 days before onset; Patients with fever or respiratory symptoms from Wuhan city and surrounding areas, or from the community with case reports, had been contacted within 14 days before the onset of the disease; there was clustering disease. (2) Clinical manifestations: ① fever and / or respiratory tract symptoms; ② imaging features of pneumonia; ③ normal or reduced leukocyte count or lymphocyte count in the early stage of the disease. Anyone with epidemiological history and any of two with clinical manifestations can be diagnosed as suspected cases. The inclusion criteria of patients (2 criteria met at the same time): (1) suspected cases of 2019 ncov pneumonia; (2) sputum, throat swab, lower respiratory tract secretion and other standards were used for real-time RT-PCR to detect 2019 ncov nucleic acid positive.

## Clinical classification of disease severity^8^

All confirmed patients were novel coronavirus pneumonia (fifth trial version) for clinical diagnosis. They were: (1) light: mild clinical symptoms, no pneumonia in imaging; (2) common pattern: fever, respiratory symptoms, imaging findings of pneumonia; (3) heavy: any of the following: ① Respiratory distress Forced, RR ≥ 30 times/min; ② in the resting state, the oxygen saturation ≤ 93%; ③ PaO2/FiO2 ≤ 300MMHG (1mmhg = 0.133kpa); (4) severe: one of the following conditions: ① respiratory failure, requiring mechanical ventilation; ② shock; ③ other organ failure requiring ICU monitoring and treatment.

### Test method

The samples of pharynx, stool and blood were collected. The blood samples were tested by the biochemical laboratory according to the operating procedures. The nucleic acids of pharynx and stool were detected by the molecular laboratory using RT-PCR method. The total RNA was extracted within 2 h, and two target genes, including orf1ab and N, were amplified and tested at the same time. Amplification conditions: reverse transcription at 42 ℃ for 5 min, pre denaturation at 95 ℃ for 10 s, denaturation at 95 ℃ for 40 cycles for 10 s, expansion at 60 ℃ and collection of fluorescence signals for 45 s. The double target detection kit was provided by Shanghai Jienuo Biotechnology Co., Ltd. (gxzz 20203400058). Results: cut off value was 40, CT value < 37 was positive, CT value > 40 was negative, 37 ~ 40 was gray area (need to be retested). In the early morning, the fasting venous blood was taken from patients for examination of blood routine, blood biochemistry, coagulation function, blood gas analysis, immunoglobulin, hypersensitive protein, procalcitonin, electrolyte, T-lymphocyte subsets, etc.

T lymphocyte subset detection kit was produced by BD Biosciences, and the flow cytometer was BD FACSCantoII. Two specimen detection tubes were prepared, 2 mL whole blood was collected using the anticoagulant tubes of ethylenediamine teacetic acid, and 100 µL whole blood was inhaled from each of the tubes into the specimen detection tubes. 1 tube to join 20 µL CD3 (FITC)/CD4 CD8 (PE) (Cy5-PE) /CD45PerCP, 2 pipe for the same type of care, respectively to join 20 µL IgG-Cy5-FITC, IgG-PE and IgG-PerCP, oscillation were conducted to mix evenly, placed in room temperature, avoid light incubation for 20 min, add 2 ml hemolysin, continue oscillation to mix evenly, placed in room temperature, avoid light incubation above 10 min, and then placed in a centrifuge, carried out in accordance with the 1500 RPM speed centrifugal, centrifugal time for 5 min, put on a clear liquid, Then add 2 ml PBS, placed in a centrifuge, carried out in accordance with the 1500 RPM speed centrifugal, centrifugal time for 5 min, put on a clear liquid, add 2 ml PBS, placed in a centrifuge, carried out in accordance with the 1500 RPM speed centrifugal, centrifugal time for 5 min, the supernatant discarded, then add in 900µL PBS, using flow cytometry instrument testing, using Cell Quest software(Becton Dickinson, San Jose, CA, USA) analysis test, the above operations were in strict accordance with the instruction to operate.

### Clinical treatment

From the date of diagnosis, the confirmed cases were given the antiviral drug lopinavir/ritonavir (200 mg/50 mg / capsule) orally, two capsule a time, three times a day; among them, the severe or critical patients were given immunoglobulin 20 g / day plus corticosteroids (40–80 mg/day methylprednisolone). According to the severity of hypoxemia, different ways and degrees of oxygen were given (low flow oxygen, high flow oxygen, nasal catheter oxygen, mask oxygen, etc.).

### Observation index

The laboratory test and imaging data were obtained from the hospital electronic medical record system as the baseline data. Laboratory examination includes: blood routine examination, blood gas analysis, coagulation function, blood biochemistry, electrolyte, bacterial and fungal culture, infection markers, T-lymphocyte subsets, etc., which are rechecked once every 3–5 days and once every 1–2 days if necessary; imaging examination mainly included chest CT examination, which was rechecked once every 3–5 days. According to previous research, focused on the observation of factors that might affect the transition from mild or common to seere or critical, such as age, body mass index, underlying diseases, white blood cells, lymphocytes, neutrophils, blood gas analysis, various inflammatory factors, Liver and kidney function, coagulation function, electrolytes, T lymphocyte subsets, etc.

### Discharge criteria^8^

The standard of discharge was no fever for at least three days, both lungs of chest CT were significantly improved, clinical remission of respiratory symptoms and negative detection of 2019-ncov nucleic acid by throat swab at least 24 h apart.

### Statistical treatment

Excel table was used to collect and sort the raw data. Spss19.0 software (Version 19.0, IBM, Chicago, IL, USA) was used for statistical analysis. The processing of missing values adopted regression estimation method. Mean ± standard deviation was used to express the normal distribution measurement data, t test was used to compare the mean of two samples, and variance test was used to compare the mean of more than two samples; median was used to describe the non normal distribution measurement data, and rank sum test was used; χ^2^ test was used to count the data; In the training set, univariate unconditional logistic regression analysis was first used to incorporate meaningful variables into the multi-factor unconditional logistic regression analysis. The Forward: LR method was used to treat the variables with significant differences in the multivariate analysis as independent risk factors, a prediction model was established; calculated the accuracy, precision, recall and F1-Score of the model, the area under the ROC curve was used to test the discrimination of the prediction model; the calibration curve was drawn according to the actual incidence and prediction incidence. The model established by the training set was validated by the data of the validation set. *P* < 0.05 was considered to be statistically significant. SPSS modeler18.0 software was used, the data of the training set established a prediction model by the random forest method, calculated the accuracy, precision, recall and F1-Score of the model; then the prediction model was used to verify in the verification set. Compare the prediction models established by the two methods of unconditional logistic regression and random forest.

## Results

### Basic characteristics

Among 408 patients in the training set, the incubation period was 2–20 days, with a median of 9 (6, 14) days. Among them, 196 cases were male (48.0%) and 212 cases were female (52.0%). The median age was 47 years (37, 56). According to China's novel coronavirus pneumonia diagnosis and treatment plan (trial version fourth) clinical classification, when 408 cases were diagnosed, 64 cases were mild, 344 cases were common type. During the 6–10 days after the diagnosis, 60 patients were seriously ill, 52 of them were severe, 8 of them were critical. Seen Table 1 for the specific clinical characteristics.

**Table 1:** Basic characteristics of COVID-19 in mild or common group and severe or critical group in training set.

Variable	All (408)	Mild or common group (348)	Severe or critical group (60)	*P*	× ^2^/Z
Gender (male%)	196 (48.0)	163 (46.8)	33 (55.0)	0.847	0.041
Incubation period	9 (6, 14)	9 (7, 14)	7 (4, 15)	0.389	− 0.862
Age	47 (37, 56)	46 (35, 56)	48 (47, 67)	0.025	− 2.236
BMI	22.84 (21.60, 24.51)	22.35 (21.05, 24.44)	26.54 (24.22, 27.34)	0.000	− 4.094
Basic diseases	164 (40.2)	130 (37.4)	34 (56.7)	0.196	1.985
Fever	296 (72.5)	236 (67.8)	60 (100)	0.007	8.039
Headache	44 (10.8)	29 (8.3)	15 (25)	0.497	0.666
Runny nose	32 (7.8)	24 (6.8)	8 (13.3)	0.213	1.447
Dry cough	116 (28.4)	98 (28.2)	18 (30.0)	0.929	0.001
Expectoration	96 (23.5)	91 (26.1)	5 (8.3)	0.095	3.168
Sore throat	64 (15.7)	58 (16.7)	6 (10.0)	0.213	1.447
Chest tightness	72 (17.6)	53 (15.2)	19 (31.7)	0.078	3.394
Flustered	32 (7.8)	17 (4.9)	15 (25.0)	0.03	4.979
Diarrhea	28 (6.9)	11 (3.2)	17 (28.6)	0.022	5.623
SP02	95 (93, 96)	95 (94, 96)	93 (84, 96)	0.015	− 2.439

In this study, 408 patients with covid-19 in the training set were divided into two groups: 348 in the light and common type group, and 60 in the heavy and critical type group. There was no significant difference between the two groups with diabetes, cardiovascular disease and chronic obstructive pulmonary disease. There were significant differences in age and BMI between the two groups (z = − 2.236, *P* = 0.025; Z = − 4.094, *P* = 0.000). In terms of clinical symptoms, fever and cough were the most common symptoms, while diarrhea, palpitation and runny nose were the less common symptoms.

### Biochemical indexes

408 patients in the training set were collected as the baseline indexes when the first pharyngeal test was positive for 2019-ncov nucleic acid. The baseline comparison between the two groups was as follows: peripheral blood lymphocyte (L) (z = − 2.305, *P* = 0.021), lactic acid (LA) (z = − 2.463, *P* = 0.014), albumin (ALB) (z = − 2.868, *P* = 0.004), Ca (z = − 1.994, *P* = 0.046), Fe (z = − 2.849, *P* = 0.004) were significant differences. There was no significant difference in leukocyte, neutrophil, hemoglobin, platelet, liver and kidney function, muscle enzyme, C-reactive protein and coagulation function. Seen Table 2 for details.

**Table 2:** Basic characteristics of COVID-19 in mild or common group and severe or critical group in training set.

Variable	All (408)	Mild and common group (348)	Severe and critical group (60)	*P*	Z
WBC	4.53 (3.22, 6.06)	4.44 (3.19, 5.91)	5.09 (4.10, 7.56)	0.078	− 1.762
N	2.83 (2.03, 4.33)	2.70 (1.93, 3.88)	3.86 (2.81, 6.44)	0.081	− 1.743
L	0.93 (0.68, 1.45)	0.97 (0.70, 1.49)	0.79 (0.58, 1.14)	0.021	− 2.305
RBC	4.46 (4.03, 4.94)	4.45 (4.07, 4.93)	4.46 (3.99, 5.04)	0.502	− 0.671
HB	136 (122, 149)	136 (122, 151)	136 (116, 149)	0.667	− 0.43
BPC	168 (126, 240)	171 (140, 245)	134 (123, 226)	0.169	− 1.375
CRP	16.27 (11.15, 30.29)	16.27 (11.00, 28.13)	16.28 (11.20, 48.70)	0.535	− 0.62
HCRP	4.74 (4.72, 4.76)	4.75 (4.73, 4.76)	4.75 (4.74, 4.76)	0.542	− 0.61
PCT	0.05 (0.04, 0.53)	0.05 (0.04, 0.05)	0.05 (0.05, 0.08)	0.164	− 1.39
PH	7.41 (7.40, 7.43)	7.41 (7.40, 7.43)	7.42 (7.40, 7.44)	0.321	− 0.993
pCO2	38 (35, 40)	38 (35, 40)	37 (33, 40)	0.493	− 0.685
pO2	84 (74, 92)	84 (77, 93)	86 (56, 89)	0.593	− 1.392
Lac	2.1 (1.9, 2.2)	2.1 (1.9, 2.2)	2.2 (1.8, 2.8)	0.408	− 0.827
Pao2	106 (87, 121)	103 (81, 116)	132 (107, 173)	0.001	− 3.251
A-AdO2	21 (5, 43)	20 (4, 39)	48 (20, 70)	0.005	− 2.83
paO2/Pao2	0.92 (0.88, 0.99)	0.92 (0.90, 1.01)	0.68 (0.63, 0.89)	0.004	− 2.91
LA	0.35 (0.09, 0.60)	0.34 (0.00, 0.57)	0.6 (0.1, 1.3)	0.014	− 2.463
HCO3	23.7 (22.6, 24.5)	23.6 (22.6, 24.4)	23.7 (23.5, 24.8)	0.587	− 0.543
SaO2	94 (93, 97)	94 (93, 96)	96 (90, 97)	0.413	− 0.818
ESR	22 (10, 42)	22 (8, 35)	30 (18, 61)	0.075	− 1.782
PT	10.4 (9.3, 11.5)	10.4 (9.0, 11.5)	10.5 (9.2, 12.1)	0.548	− 0.6
PT (%)	129.92 (118, 142)	129.92 (129.92, 142.5)	129 (125, 130)	0.605	− 0.517
INR	0.93 (0.78, 1.05)	0.93 (0.77, 1.04)	0.92 (0.79, 1.09)	0.684	− 0.406
APTT	30.90 (27.67, 33.90)	30.50 (27.60, 33.80)	31.80 (28.67, 35.70)	0.314	− 1.006
TT	14.10 (13.27, 14.92)	14.04 (12.89, 14.90)	14.43 (13.90, 15.40)	0.143	− 1.465
FIB	3.12 (2.63, 4.73)	3.12 (2.61, 4.74)	3.52 (2.81, 4.61)	0.62	− 0.496
FDP	1.66 (1.36, 1.87)	1.66 (1.36, 1.87)	1.65 (1.38, 2.49)	0.419	− 0.809
TB	10.1 (7.9, 16.4)	10.1 (8.1, 15.4)	11.3 (7.0, 19.3)	0.951	− 0.061
ALT	22.2 (15.5, 34.0)	22.3 (16.0, 36.0)	20.0 (14.7, 28.8)	0.347	− 0.94
AST	27 (21, 34)	27 (22, 34)	25 (19, 34)	0.603	− 0.52
GGT	27 (17, 41)	27.4 (17.2, 38.8)	36.8 (17.9, 63.4)	0.395	− 0.85
ALP	66 (54, 80)	66 (54, 81)	66 (52, 70)	0.461	− 0.737
ALB	41.1 (36.7, 46.0)	41.9 (38.6, 46.8)	36.5 (34.3, 40.2)	0.004	− 2.868
GLO	29.5 (26.0, 32.6)	29.6 (25.9, 31.7)	28.2 (26.0, 36.4)	0.464	− 0.732
PALB	235 (210, 263)	236 (211, 261)	245 (188, 268)	0.806	− 0.246
TBA	5.3 (3.1, 6.7)	5.4 (3.1, 6.7)	5.2 (2.6, 7.0)	0.921	− 0.099
LDH	230 (186, 292)	228 (184, 281)	256 (187, 373)	0.212	− 1.247
HBDH	152.4 (118.9, 179.5)	160.5 (160.5, 160.5)	160.5 (160.5, 165.3)	0.485	− 0.698
CK	82.8 (60.5, 107.6)	86.6 (62.2, 109.2)	64.7 (56.8, 100.10)	0.251	− 1.148
MYO	20.58 (16.28, 27.94)	20.40 (16.50, 27.58)	21.94 (14.54, 61.50)	0.398	− 0.846
CTnT	0.01 (0.01, 0.02)	0.01 (0.00, 0.01)	0.01 (0.00, 0.01)	0.521	− 0.641
CK-MB	2.13 (0.41, 5.82)	2.18 (0.41, 5.90)	1.15 (0.10, 3.64)	0.127	− 1.526
D-Dimer	0.63 (0.55, 0.63)	190.0 (0.63, 420.0)	135.0 (0.49, 610.0)	0.846	− 0.194
BUN	3.5 (2.9, 4.4)	3.5 (3.0, 4.3)	3.5 (2.9, 5.6)	0.62	− 0.496
CR	57.8 (49.1, 68.9)	57.2 (48.4, 67.9)	66.4 (52.0, 86.9)	0.126	− 1.531
UA	254.5 (209.0, 335.9)	267.8 (214.1, 333.5)	236.5 (180.5, 381.6)	0.795	− 0.26
GLU	6.7 (5.6, 7.9)	6.7 (5.4, 7.8)	6.6 (6.0, 8.6)	0.839	− 0.203
CA	2.16 (2.03, 2.33)	2.20 (2.06, 2.34)	2.03 (1.97, 2.23)	0.046	− 1.994
MG	0.905 (0.843, 0.947)	0.901 (0.843, 0.954)	0.914 (0.742, 0.944)	0.917	− 0.104
P	1.00 (0.84, 1.12)	1.00 (0.84, 1.13)	0.99 (0.86, 1.07)	0.567	− 0.572
FE	16.9 (13.3, 20.8)	17.3 (14.0, 21.5)	13.4 (7.1, 16.9)	0.004	− 2.849
K	4.05 (3.80, 4.35)	4.06 (3.81, 4.37)	3.88 (3.64, 4.25)	0.267	− 1.11
NA	140.8 (139.0, 142.9)	140.8 (139.3, 143.2)	139.5 (134.1, 142.2)	0.061	− 1.871
CL	101.2 (98.4, 104.9)	101.1 (98.5, 104.8)	101.3 (98.2, 105.6)	0.817	− 0.232
IGG	13.03 (11.47, 14.20)	13.01 (11.47, 14.12)	13.87 (11.27, 14.86)	0.481	− 0.704
IGA	2.14 (1.64, 2.66)	2.14 (1.62, 2.67)	2.18 (1.69, 2.48)	0.62	− 0.495
IGM	1.22 (0.61, 1.58)	1.22 (0.56, 1.55)	1.21 (0.87, 2.39)	0.219	− 1.228
CHOE	9397 (8559, 10,214)	9526 (8752, 10,503)	8955 (7606, 9240)	0.01	− 2.584
NT-proBNP	50 (50, 50)	50 (20, 50)	50 (50, 442)	0.008	− 2.651
Lopinavir / ritonavir	133 (32.59)	110 (31.61)	23 (38.33)	0.568	0.356
Abidor	141 (34.56)	124 (35.63)	17 (28.33)	0.663	0.178
Combination of two	134 (32.84)	114 (32.76)	20 (33.33)	0.770	0.193

WBC, white blood cell (× 10^9^/L); N, neutrophils (× 10^9^/L); L, lymphocyte (× 10^9^/L); RBC, red blood cell (× 10^12^/L); HB, hemoglobin (g/L); BPC, blood platelet (× 10^9^); CRP, C-reactive protein(mg/L) ; PCT, procalcitonin (ug/L); LA, lactic acid (mmol/L); ESR, erythrocyte sedimentation rate (mm/h); PT, prothrombin time; INR, international standardized ratio; APTT, partial prothrombin time; TT, thrombin time; FIB, Fibrinogen (g/L); FDP, Fibrinogen degradation products (mg/L); TB, total bilirubin (umol/l); ALT, alanine aminotransferase (U/L); AST, aspartate aminotransferase (U/L); GGT, glutamyl transpeptidase (U/L); ALP, alkaline phosphatase (U/L); ALB, albumin (g/L); GLO, globulin (g/L); PALB, prealbumin (g/L); TBA, total bile acid (umol/L); LDH, lactate dehydrogenase (U/L); HBDH, α-hydroxybutyrate dehydrogenase (U/L); CK, creatine kinase (U/L); MYO, myoglobin; CTnT, troponin (ng/ml) ; CK-MB, creatine kinase isoenzyme; BUN, urea nitrogen (mmol/L); CR, creatinine (umol/L); UA, uric acid (umol/L); GLU, blood glucose (mmol/L); CA, calcium (mmol/L); MG, magnesium (mmol/L); P, phosphorus (mmol/L); FE, iron (umol/L); K, potassium (mmol/L); NA, sodium (mmol/L); CL, chlorine (nnol/L); IGG, immunoglobulin G (g/L); IGA, immunoglobulin A(g/L) ; IGM, immunoglobulin M (g/L); CHOE, cholinesterase (U/L).

In terms of antiviral treatment of 348 in the light and common type group, 110 (31.61%) patients were treated with lopinavir / ritonavir, 124 (35.63%) with abidor, 114 (32.76%) with combination of the two; of 60 in the heavy and critical type group, 23 (38.33%) with lopinavir / ritonavir, 17 (28.33%) with abidor and 20 (33.33%) with combination of the two. There was no significant difference in the use of antiviral drugs between the two groups. Seen Table 2.

### T-lymphocyte subsets

In the training set, T-lymphocyte subsets were detected when the two groups were diagnosed. The counts of CD3^+^ T cells (z =5.621, *P* = 0.000), CD4^+^ T cells (z = − 5.617, *P* = 0.000), CD8^+^ T cells (z = − 5.456, *P* = 0.000) in the severe and critical groups were significantly lower than those in the light and general groups. But there was no significant difference between CD3^+^/CD45^+^, CD4^+^/CD45^+^, CD4^+^/CD8^+^ in the two groups. Seen Table 3 for specific results.

**Table 3:** The feature of T-lymphocyte subsets in mild or common group and severe or critical group in training set.

Variable	All (408)	Mild or common group (348)	Severe or critical group (60)	*P*	Z
CD3^+^ (PCs/ul)	1024 (701, 1236)	1044 (792, 1252)	160 (154, 529)	0.000	− 5.621
CD3^+^/CD45^+^ (%)	67 (58, 73)	68 (62, 73)	30 (30, 54)	0.071	− 1.804
CD4^+^ (PCs/ul)	524 (404, 669)	548 (447, 766)	72 (70, 273)	0.000	− 5.617
CD4^+^/CD45^+^ (%)	39 (31, 46)	40 (34, 47)	14 (14, 33)	0.089	− 1.701
CD8^+^ (PCs/ul)	380 (233, 469)	413 (279, 532)	79 (79, 211)	0.000	− 5.456
CD8^+^/CD45^+^ (%)	24 (20, 27)	24 (22, 30)	15 (15, 17)	0.175	− 1.355
CD4^+^/CD8^+^	1.50 (1.00, 2.00)	1.50 (1.30, 2.00)	0.90 (0.90, 1.40)	0.590	− 0.539

### Dynamic changes of T-lymphocyte subsets

**Figure 1:**
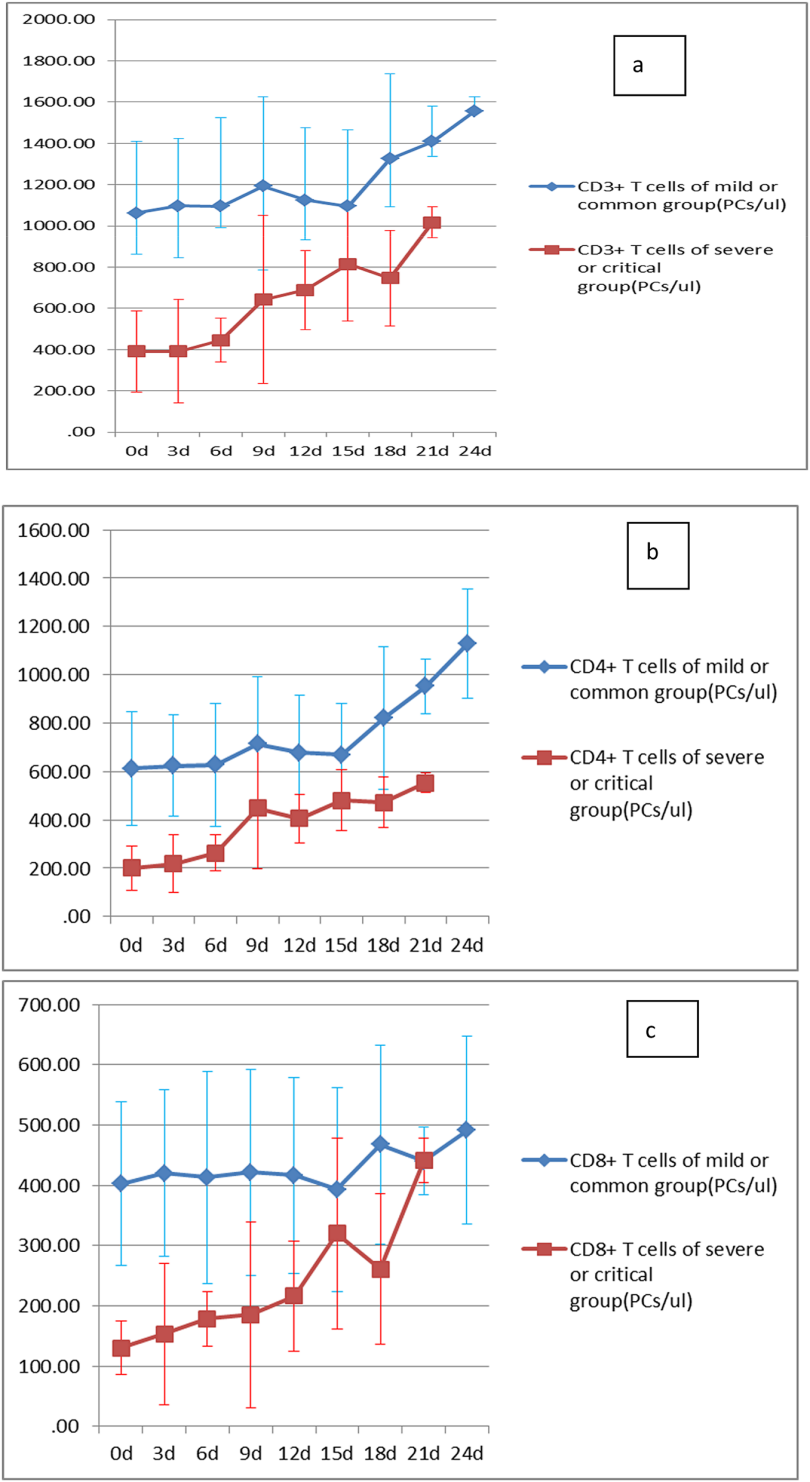
T-lymphocyte subsets dynamic changes of in training set: dynamic changes of CD3^+^ T cells (a), dynamic changes of CD4^+^ T cells (b), dynamic changes of CD8^+^ T cells (c).

The counts of CD3^+^, CD4^+^, CD8^+^ T-lymphocytes in the training set were rechecked every 3–4 days. The dynamic changes of T-lymphocyte subsets in the two groups were shown in 1A, 1B and 1C of Fig. 1. It could be seen that the CD3^+^, CD4^+^, CD8^+^ T lymphocytes in the severe and critical groups were significantly lower than those in the light and common groups at all time points, and the CD3^+^, CD4^+^, CD8^+^ T lymphocytes in the severe and critical groups recovered slowly with the improvement of the condition.

### Univariate and multivariate regression analysis

In the training set, L, LA, ALB, CA, Fe, CD4^+^ and CD8^+^ T cells were selected for univariate and multivariate regression analysis. The results showed that LA and CD8^+^ T cells were independent factors (*P* = 0.042, or = 1097.983, 95% CI 1.303, 924,798.262; *P* = 0.010, or = 0.903, 95% CI 0.835, 0.975), which could predict the transition from mild and common patients to severe and critical patients in the early stage. The cut off values were 2.05 and 190 respectively. Seen Table 4 for details.

**Table 4:** Univariate and multivariate analysis of the transition from light or ordinary to heavy or critical in training set.

	B	S.E	Exp (B)	95% of EXP(B)C.I		P	B	S.E	Exp(B)	95% of EXP(B) C.I		P
				Low limit	Upper limit					Low limit	Upper limit	
L	− 1.182	0.685	0.307	0.08	1.175	0.085						
LA	0.683	0.506	1.981	0.734	5.343	0.177	7.001	3.437	1097.98	1.304	924,798.262	0.042
ALB	− 0.143	0.052	0.866	0.782	0.959	0.006						
CA	− 1.425	1.034	0.241	0.032	1.827	0.168						
FE	− 0.137	0.049	0.872	0.792	0.961	0.006						
CD4^+^	− 0.029	0.009	0.972	0.955	0.989	0.001						
CD8^+^	− 0.044	0.014	0.957	0.931	0.984	0.002	− 0.102	0.04	0.903	0.836	0.976	0.01

### ROC curve

**Figure 2:**
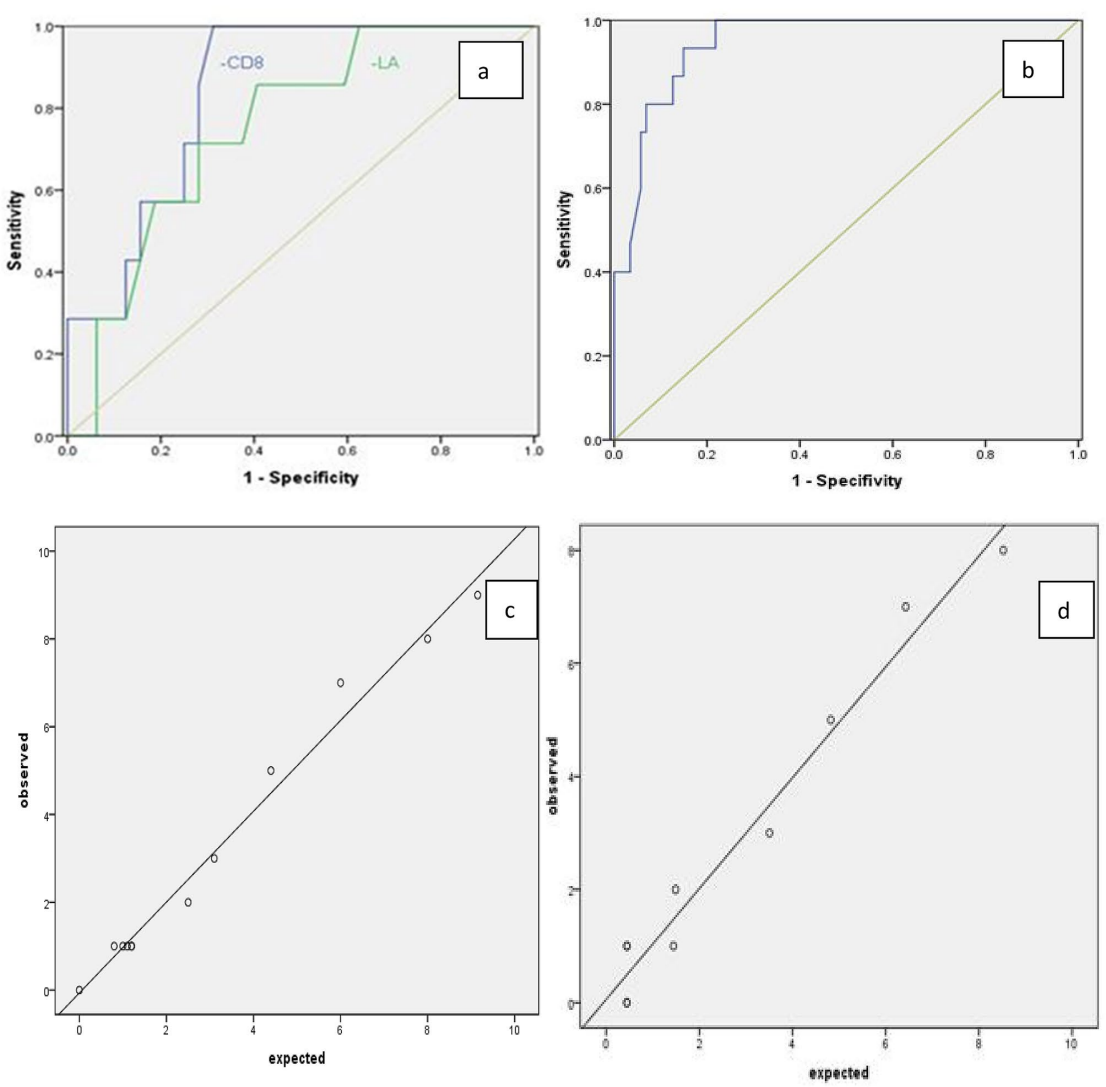
ROC curve of independent influence factors La and CD8^+^ T cells in training set (a) and ROC curve of CD8^+^  T cells in validation set (b). Fitting curve of actual observation value and prediction model value of prediction model established by CD8^+^ T cells in training set (c) and in validation set (d).

Two independent factors, LA and CD8^+^ T cells, were used to draw ROC curve. The area under LA curve was 0.754 (0.581, 0.928), and the area under CD8^+^ T cell curve was 0.842 (0.713, 0.970). The sensitivity and specificity of LA were 0.857 and 0.594, accuracy, precision, recall and F1-score were 0.912, 0.750, 0.601 and 0.667; The sensitivity and specificity of CD8^+^ T cells were 0.959 and 0.687, accuracy, precision, recall and F1-score were 0.923, 0.833, 0.714 and 0.769. Seen 2a of Fig. 2 for ROC curve.

### In the training set, draw calibration curve according to the actual observation value and prediction model value

Seen 2c of Fig. 2, and the actual observation value and prediction model value were highly consistent.

### Validation of the model

CD8^+^ T cells in the training set were independent factors for early prediction of the transition from light and common patients to heavy and critical patients, and the volume under the ROC curve was 0.842 (0.713, 0.970). The area under ROC curve was to be used to evaluate the differentiation of clinical prediction model of training set in external verification.

A total of 78 novel coronavirus pneumonia patients were admitted to the Third People's Hospital of Kunming as a validation set. The basic characteristics of the validation set were shown in supplementary Table 1, and the data of the validation set and the training set were comparable. According to the regression equation established by the training set, the prediction probability of the verification set was calculated and the ROC curve was drawn, as shown 2 b of Fig. 2. AUC of ROC curve was 0.906 (0.861, 0.981). The prediction model obtained good identification ability by external verification in the validation set as well as the training set. The calibration curve for actual observation value and prediction model value of validation set was drawn, as shown 2d of Fig. 2. The actual observation value was highly consistent with the prediction model value.

### Random forest model

**Figure 3:**
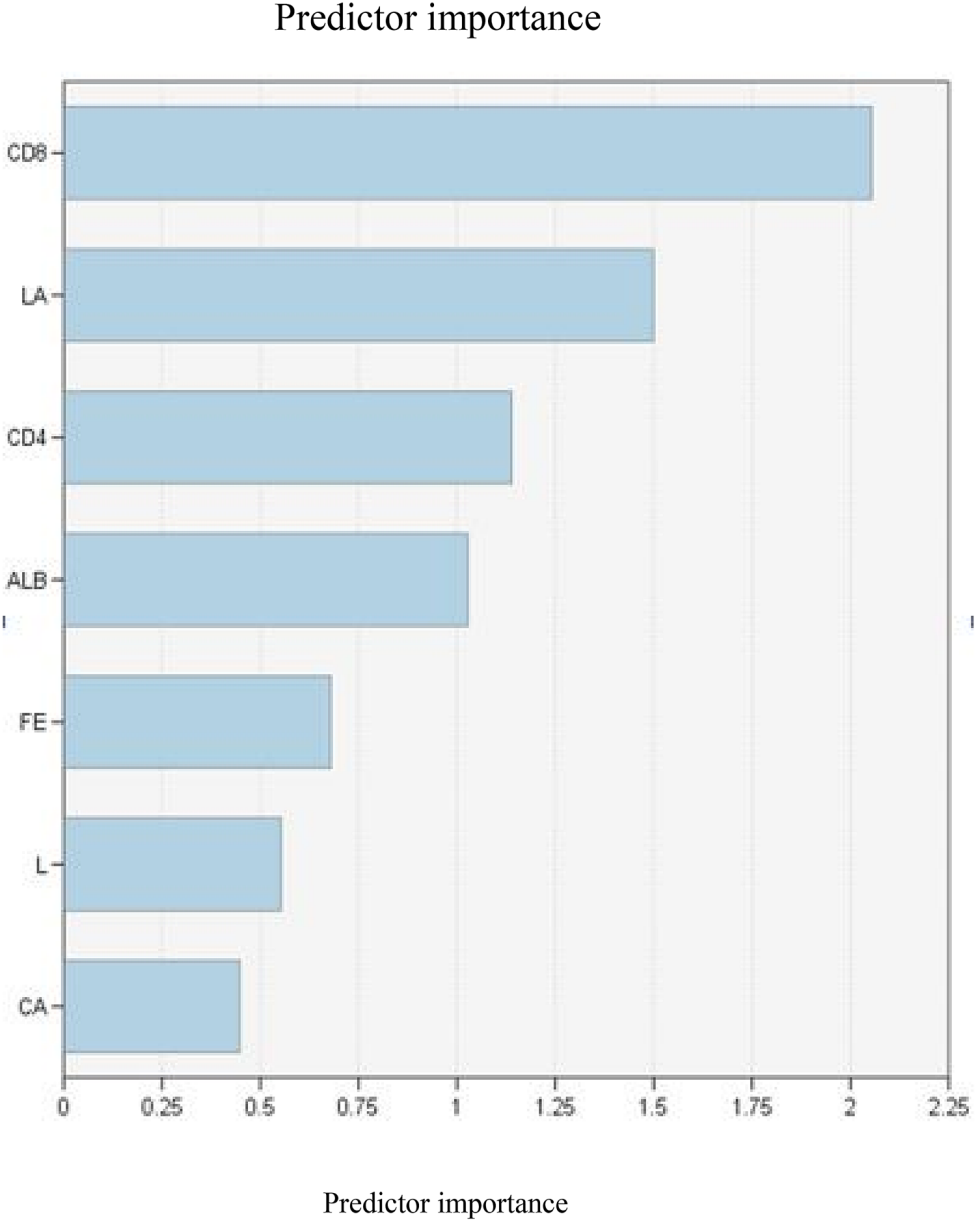
Random forest predictor ordering.

In the training set, the statistically significant variables of single factor analysis were included in the random forest model. The number of preselected variables at each tree node in the forest was set to the square root of all variables, and the total number of trees was set to 500. The analysis results showed that the importance of each variable in descending order was: CD8^+^ T cells, lactic acid, CD4^+^, ALB, FE, L, CA. Seen Fig. 3. Accuracy, precision, recall and F1-score of the random forest model were 0.948, 0.917, 0.786 and 0.845. The random forest model was substituted into the verification set data for verification, accuracy, precision, recall and F1-score were 0.935, 0.901, 0.714, 0.801.

### Prognosis

All the patients were treated by comprehensive treatment, in the training set, only 2 cases died and others discharged from hospital; in the validation set, all the patients were cured. All The average length of stay was 15.31 ± 5.64 days in 348 light and general patients, and 22.76 ± 4.82 days in 60 severe and critical patients. There was no significant difference in the average hospitalization days between the light and common type patients in the validation set and the light and common type patients in the training set (t = 0.732, *P* = 0.466); there was no significant difference in the average hospitalization days between the heavy and critical type patients in the validation set and the heavy and critical type patients in the training set (t = 0.046, *P* = 0.964). The training set and the validation set were comparable in the length of stay. Seen supplementary Table 2 for details.

## Discussion

Since the outbreak of 2019 ncov infection in December 2019, there were 2000 to 4000 newly diagnosed patients every day in China, with a large number of serious cases. With the global spread of covid-19, the number of deaths had increased significantly. If severe cases before they transferred to severe could be identified as early as possible, and necessary interventions could be carried out as early as possible, the mortality rate might be reduced.

Most of the patients with early onset of covid-19 were not very serious, most of them were light and common type, a few of them were heavy / critical type, and a few of them were asymptomatic carriers. But in the course of treatment, some light and common patients would rapidly change into heavy and critical, and develop into severe pneumonia and acute respiratory failure, even death. Some studies had shown that the elderly with 2019 ncov infection were prone to severe or critical diseases because they were mostly combined with other basic diseases, and the mortality rate would also be high^4,9,10^. At the same time, the decrease of lymphocyte count was related to the progress of the disease, and the lymphocyte related index might be a potential predictor^11^.

The data of the training set in this study were all light or common type at the time of diagnosis. In the process of giving oxygen inhalation, monitoring vital signs and antiviral treatment at the same time, within 3–10 days of admission, 60 patients' condition suddenly increased without any sign, and had to be transferred to ICU for rescue. Comparing the baseline indexes of 60 patients before aggravation with that of 348 patients without disease change, there might be early warning factors of aggravation. The BMI, L, LA, blood calcium, CD3^+^, CD4^+^ and CD8^+^ T cell counts of the two groups were significant before the disease change, but only LA and CD8^+^ T cell counts were independent risk factors for the disease change. It showed that LA and CD8^+^ T cells had changed a lot before the aggravation of the disease, and they were independent risk factors that affected the development of light and common patients to heavy and dangerous ones. Further ROC curve analysis showed that the area under LA curve was 75.4%, the sensitivity was 85.7%, and the specificity was 59.4%; while the area under CD8^+^ T cell curve was 84.2%, the sensitivity was 95.9%, and the specificity was 68.7%. The fitting validity of the prediction model established by CD8^+^ T cells was also consistent. It showed that serum LA and CD8^+^ T cell count could not only change before the exacerbation of patients with mild and common new coronary pneumonia, but also predict the occurrence of exacerbations of patients, and its prediction effect was better. For the early intervention of medical staff in time, to prevent the progress of the disease to get time.

The prediction model established by 408 patients in the First People's Hospital of Zaoyang was verified in 78 light and common patients in the Third People's Hospital of Kunming. The area under the CD8^+^ T cell curve of the validation set was 90.6%, that under the CD8^+^ T cell curve of the contrast training set was 84.6%, and that under the curve was more than 70%, indicating that the prediction model had a good discrimination ability. Generally speaking, due to the different baseline characteristics of patients in different medical centers and different levels of medical treatment, the area under the ROC curve of the external validation set will generally decrease or increase, but the fluctuation range was within 10% clinically acceptable. In the training set, the scatter points fluctuated around the reference line, and the separation of the scatter points did not deviate from the reference line significantly, which suggested that the predicted observation value of the clinical prediction model was consistent with the actual observation value. At the same time, when the prediction model was applied to another medical center, the scattered points of curve fitting also fluctuated around the reference line, which showed that the prediction model had good accuracy and stability.

At the same time, using the random forest method to analyze the training set data again, the established model and verification in the verification group, all indicators were better than the unconditional logistic regression model, and the importance of each variable could be analyzed from high to low, the order was: CD8^+^ T cells, lactate, CD4^+^, ALB, FE, L, CA. It showed that the random forest had good adaptability to complex data, could give the ranking of each variable under high prediction accuracy, and improved the efficiency of the test.

It should be noted that the dynamic changes of CD4^+^ and CD8^+^ T lymphocytes were also valuable for the prognosis of the disease. With the improvement of the condition, CD4^+^ and CD8^+^ T lymphocytes gradually recovered in severe and critical patients. These data supported previous studies that lymphopenia, and in particular low T cell counts, were correlated with severe disease, but this study added that measuring T cell counts at disease onset (while still mild in all patients) could predict the outcome or disease progression and was therefore useful for patient management.

Some study thought^12^ that the reduction of CD8^+^ T cells in peripheral blood was closely related to serious diseases, and it had been determined that T cell apoptosis and migration to inflamed tissues were possible mechanisms that drived the reduction of peripheral T lymphocytes, and severe COVID- 19 patients were characterized by extensive T cell loss and subsequent T cell proliferation. Another study suggested^13^ that the immune characteristics of COVID-19 hospitalized patients were heterogeneous, and their CD8^+^ T cell exhaustion and disease severity might be related to changes over time. The cellular origin (T cells, dendritic cells, or macrophages) of inflammatory cytokine storms in novel coronavirus pneumonia patients remains to be determined. Whether the sharp decline or even depletion of CD4^+^, CD8^+^ T cells will affect the replication or elimination of the virus remains to be further studied.

The sample of this study was too small, needs to be confirmed by a large sample. The patients were only from 2 hospitals and entirely from China, which could potentially limit the generalizability in other areas of the world. However, our focus was to identify the risks of patients early and carry out the necessary interventions in time to reduce the occurrence of critically ill patients and the decline in mortality.

## Supplementary Information

Supplementary Information.
