# Supplementary material for: CD8+ T cells predicted the conversion of common covid-19 to severe
Source: Sci Rep. 2021 Jan 26;11:2169. doi: 10.1038/s41598-021-81732-4 (PMC7838185; doi:10.1038/s41598-021-81732-4)
Supplement: Supplementary file 1 — Supplementary Information. [file 41598_2021_81732_MOESM1_ESM.docx]

**CD8^+^T cells predicted the conversion of common covid-19 to severe**

Li Liu^1^, Zhiyong Chen^2^, Yingrong Du^1^, Jianpeng Gao^1^, Junyi Li^1^, Tiqin Deng^2^, Chen Chen^2^, Lin Wang^1^, Yongrui Yang^1^, Chunyun Liu^1^

**Supplementary table 1 Baseline characteristics of biochemical indicators of COVID-19 in mild or common group and severe or critical group in validation set**

| variable | all | mild or common group（64） | severe or critical group（14） | P | Z/*X*^2^ |
| --- | --- | --- | --- | --- | --- |
| WBC | 4.74(3.59,6.02) | 4.73（3.45，6.15） | 4.94(4.39,5.99) | 0.661 | -0.439 |
| N | 2.81(2.20,3.86) | 2.64（2.09，3.75） | 3.32(2.85,4.92) | 0.073 | -1.793 |
| L | 1.40(0.93,1.65) | 1.45（1.02，1.73） | 0.86(0.58,1.15) | 0.001 | -2.58 |
| RBC | 4.73(4.31,5.32) | 4.72（4.31，5.41） | 5.04(4.00,5.10) | 0.583 | -0.549 |
| HB | 143(130,157) | 143（129，157） | 142(130,158) | 0.869 | -0.165 |
| BPC | 226(169,270) | 240（169，285） | 172(134,244) | 0.182 | -1.336 |
| CRP | 16.27(15.60,16.28) | 16.27（15.77，16.28） | 16.28(14.50,16.28) | 0.72 | -0.358 |
| HCRP | 4.75(1.05,6.10) | 4.75（0.72，5.73） | 4.75(4.75,12.16) | 0.091 | -1.69 |
| PCT | 0.05（0.05，0.05） | 0.05(0.05,0.05) | 0.05(0.05,0.07) | 0.002 | -3.063 |
| PH | 7.40(7.38,7.42) | 7.40(7.38,7.41) | 7.40(7.40,7.44) | 0.58 | -0.553 |
| pCO2 | 39(36,41) | 39(36.3,40.8) | 39(34,41) | 0.927 | -0.092 |
| pO2 | 80（71，86） | 77(70,84) | 86(76,89) | 0.164 | -1.392 |
| Lac | 2.0（1.7，2.6） | 1.9(1.7,2.5) | 2.6(2.1,3.1) | 0.037 | -2.091 |
| Pao2 | 77（69，121） | 73(69,112.5） | 136(115,181) | 0.007 | -2.696 |
| A-AdO2 | 8（－2，44） | 3(-5.5,34.3) | 44(20,53) | 0.01 | -2.563 |
| paO2/Pao2 | 0.89（0.64，1.03） | 0.96(0.71,1.07) | 0.67(0.63,0.88) | 0.027 | -2.215 |
| LA | 0.1（0.00，0.60） | 0(-0.1,0.46) | 0.5(0.1,0.6) | 0.033 | -2.138 |
| HCO3 | 23.7（22.6，25.4） | 23.7(22.4,25.4) | 23.7(23.5,25.4) | 0.985 | -0.018 |
| SaO2 | 95（93，97） | 95(93,96.7) | 96(95,97) | 0.191 | -1.309 |
| ESR | 12（5，28） | 8.50(4.25,21.11) | 18.46(15,61) | 0.038 | -2.072 |
| PT | 8.92（8.11，9.76） | 8.94(8.05,9.76) | 9.22(8.09,10.54) | 0.558 | -0.586 |
| PT(%) | 129.92（118，142） | 129.92(118,142.5) | 125(109,142) | 0.583 | -0.549 |
| INR | 0.77(0.70,0.83) | 0.76(0.69,0.83) | 0.79(0.70,0.89) | 0.533 | -0.623 |
| APTT | 28.48(26.34,31.21) | 28.04(25.96,30.39) | 30.65(26.34,34.25) | 0.227 | -1.208 |
| TT | 14.45(13.92,15.45) | 14.55(13.83,15.36) | 14.43(13.98,17.41) | 0.855 | -0.183 |
| FIB | 2.92(2.60,3.52) | 2.80(2.57,3.25) | 3.52(2.90,4.61) | 0.1 | -1.647 |
| FDP | 1.87(1.19,1.87) | 1.87(1.11,1.87) | 1.87(1.38,2.49) | 0.332 | -0.97 |
| TB | 12.4(8.5,20.8) | 12.4(8.5,21.3) | 12.1(8.1,19.3) | 0.714 | -0.366 |
| ALT | 18.6(12.5,27.1) | 19.4(12.8,26.8) | 15.9(12.0,34.1) | 0.647 | -0.458 |
| AST | 22(16,27) | 22(16,27) | 19(16,30) | 0.7 | -0.385 |
| GGT | 25(16,36) | 23.9(15.6,32.4) | 38(14.2,65.2) | 0.143 | -1.464 |
| ALP | 68(58,91) | 70(59,93) | 56(47,85) | 0.153 | -1.428 |
| ALB | 39.1(36.0,48.8) | 41.3(36.7,48.6) | 35.6(29.9,48.8) | 0.1 | -1.647 |
| GLO | 31.0(28.4,33.6) | 30.7(28.5,32.7) | 33.4(26.0,36.4) | 0.305 | -1.025 |
| PALB | 239(187,292) | 248(188,295) | 225(153,278) | 0.351 | -0.933 |
| TBA | 4.5（2.6，6.6） | 4.3(2.5,5.8) | 6.1(4.9,10.2) | 0.048 | -1.978 |
| LDH | 191（153，228） | 188(148,227) | 203(153,256) | 0.234 | -1.19 |
| HBDH | 152.4（118.9，179.5） | 151.9(119.2,179.2) | 165.3(111.3,220.0) | 0.634 | -0.476 |
| CK | 73.0(57.8,97.4) | 77.5(60.9,101.5) | 60.7(42.2,92.20) | 0.213 | -1.245 |
| MYO | 17.64（14.54，27.58） | 18.97(16.11,27.85) | 14.91(14.43,21.94) | 0.487 | -0.695 |
| CTnT | 0.01（0.01，0.02） | 0.01(0.01,0.02) | 0.01(0.01,0.01) | 0.447 | -0.761 |
| CK-MB | 0.34（0.13，0.77） | 0.38(0.17,0.76) | 0.10(0.09,0.92) | 0.234 | -1.19 |
| D-Dimer | 0.63（0.55，0.63） | 0.63(0.61,0.63) | 0.49(0.40,1.32) | 0.876 | -0.156 |
| BUN | 3.2(2.8,4.0) | 3.3(2.8,3.8) | 3.0(2.9,5.6) | 0.714 | -0.367 |
| CR | 54.2（44.1，73.1） | 53.9(44.8,71.6) | 63.2(41.1,78.1) | 0.913 | -0.11 |
| UA | 295.1(191.4,366.7) | 295.6(211.9,360.8) | 257.3(168.0,381.6) | 0.798 | -0.256 |
| GLU | 6.2(5.7,6.7) | 6.2(5.3,6.7) | 6.6(6.1,6.9) | 0.12 | -1.555 |
| CA | 2.28(2.09,2.46) | 2.31(2.18,2.48) | 2.03(1.99,2.23) | 0.022 | -2.288 |
| MG | 0.861(0.789,1.037) | 0.856(0.793,0.939) | 0.944(0.731,1.048) | 0.898 | -0.128 |
| P | 0.96(0.82,1.07) | 0.98(0.82,1.07) | 0.91(0.79,1.07) | 0.558 | -0.586 |
| FE | 12.9(6.5,22.7) | 13.9(8.3,24.5) | 7.1(4.5,7.90 | 0.031 | -2.159 |
| K | 4.19(3.77,4.40) | 4.20(3.80,4.42) | 3.88(3.71,4.25) | 0.4 | -0.842 |
| NA | 140.6(138.4,142.1) | 140.7(138.5,142.1) | 139.0(134.0,142.2) | 0.4 | -0.842 |
| CL | 104.2(101.4,106.1) | 104.2(101.4,105.9) | 103.4(99.1,107.3) | 0.855 | -0.183 |
| IGG | 11.84(10.28,14.86) | 12.08(10.39,14.86) | 11.73(9.89,14.86) | 0.798 | -0.256 |
| IGA | 1.79(1.26,2.41) | 1.69(1.25,2.65) | 2.03(1.56,2.20) | 0.742 | -0.329 |
| IGM | 1.26(0.98,1.64) | 1.26(0.81,1.74) | 1.21(0.98,1.58) | 0.985 | -0.018 |
| CHOE | 8955(7449,10050) | 9113(7703,10071) | 8532(4259,9228) | 0.2 | -1.281 |
| NT-proBNP | 50（50，50） | 50(50,50) | 50(50,164) | 0.063 | -1.858 |
| Lopinavir / ritonavir | 26（33.33） | 20（31.25） | 6（42.85） | 0.555 | 0.348 |
| Abidor | 24（30.76） | 20（31.25） | 4(28.57) | 0.889 | 0.019 |
| combination of two | 28（35.89） | 24（37.50） | 4（38.57） | 0.675 | 0.183 |

**Supplementary table 2 Comparison of hospitalization days of patients in training set and verification set**

|  | training set | verification set | t | p |
| --- | --- | --- | --- | --- |
| Days of hospitalization for mild or common group | 15.31±5.64 | 16.16±5.89 | 0.732 | 0.466 |
| Days of hospitalization for severe or critical group | 22.76±4.82 | 22.86±4.63 | 0.046 | 0.964 |
